# A homozygous *PRDX3* pathogenic variant in a paediatric case of spinocerebellar ataxia type 32

**DOI:** 10.1007/s10048-025-00869-w

**Published:** 2025-12-06

**Authors:** Jiaxuan Yang, Yonglin Yu, Hongfang Jiang, Yueping Che, Dingwen Wu, Haifeng Li, Yaoqin Hu, Jinpiao Zhu, Daqing Ma

**Affiliations:** 1 Perioperative and Systems Medicine Laboratory, Children’s Hospital, National Clinical Research Centre for Child Health, Zhejiang University School of Medicine, Hangzhou, 310015 China; 2 Department of Anaesthesiology, Children’s Hospital, Zhejiang University School of Medicine, Hangzhou, 310015 China; 3 Department of Rehabilitation, Children’s Hospital, Zhejiang University School of Medicine, Hangzhou, 310015 China; 4 Department of Genetics and Metabolism, Children’s Hospital, Zhejiang University School of Medicine, Hangzhou, 310015 China; 5 Division of Anaesthetics, Pain Medicine & Intensive Care, Department of Surgery and Cancer, Faculty of Medicine, Imperial College London, Chelsea and Westminster Hospital, London, SW10 9NH UK

**Keywords:** PRDX3, Spinocerebellar ataxia type 32, SCAR32, Cerebellar atrophy

## Abstract

Spinocerebellar ataxia type 32 (SCAR32) is a rare autosomal neurodegenerative disorder caused by mutations in the peroxiredoxin 3 (*PRDX3*) gene, which encodes a mitochondria-specific antioxidant enzyme critical for maintaining cellular redox homeostasis. Here, we reported a case of a homozygous nonsense mutation (c.619 C > T; p.Arg207*) in *PRDX3* of a 12-year-old Chinese boy. This mutation was predicted to result in premature termination of protein translation. Consequently, the patient presented with slowly progressive gait ataxia and cerebellar vermis atrophy. Mild cognitive impairment was also observed, with deficits in perceptual reasoning and processing speed. Additionally, he showed increased thyroid autoantibodies and thyroid enlargement. This case broadens the phenotypic spectrum of *PRDX3*-related disease, highlights its genetic and clinical heterogeneity, and reinforces the importance of early genetic testing in paediatric ataxia.

## Introduction

Hereditary cerebellar ataxia (HCA) is a group of neurodegenerative disorders characterized by motor coordination deficits and cerebellar atrophy [1]. In 2021, Rebelo et al. [2] first identified bi-allelic loss-of-function mutations in the peroxiredoxin 3 (*PRDX3*) gene, which resulted in the autosomal recessive spinocerebellar ataxia type 32 [SCAR32, Online Mendelian Inheritance in Man (OMIM) #619648], typically presented with non-progressive cerebellar ataxia. Recently, Naef et al. further demonstrated the *PRDX3* mutational spectrum with identifying c.525_535delGTTAGAAGGTT (p.Leu176TrpfsTer11) and c.425 C >G (p.Ala142Gly) [3]. In the present study, we identified a homozygous pathogenic variant in the *PRDX3* gene in a young Chinese patient presenting with gait ataxia, cerebellar vermis atrophy, and increased thyroid autoantibodies. Additionally, we reviewed previously reported SCAR32 cases to further validate and broaden the clinical spectrum of this rare disorder. These findings underscore the importance of early genetic diagnosis in ataxia and highlight the need for comprehensive, multidisciplinary management strategies tailored to the diverse manifestations of SCAR32.

## Methods

The study was approved by the Ethics Committee of Children’s Hospital, Zhejiang University School of Medicine (2025-IRB-0126-P-01). Peripheral blood samples from the proband and his parents were collected for whole-exome sequencing (WES). The resulting reads were aligned to the human reference genome (hg19, UCSC database), allowing for assessment of target region coverage and sequencing quality. Variants with a minimum coverage of 10× were analysed using bioinformatics tools and evaluated for pathogenicity according to the guidelines of the American College of Medical Genetics and Genomics (ACMG). Variant nomenclature followed the recommendations of the Human Genome Variation Society (HGVS) (http://www.hgvs.org/mutnomen/).

## Results

### Case presentation

A 12-year-old Chinese boy (36.0 kg, 147 cm) born to consanguineous parents developed progressive gait abnormalities starting at age 10. His parents and his older brother were unaffected. His initial symptoms included torso instability, foot eversion, and hip internal rotation during walking. Over two years, gait disturbances progressively worsened.

Physical examination revealed gait ataxia, a positive finger-to-nose test, and knee hyperreflexia. Muscle strength and tone were preserved, and the Babinski sign was negative. Postural asymmetry (lower left shoulder, leftward head tilt) was observed due to truncal imbalance, without clinical features of cervical dystonia. Internally rotated hips, valgus knees, and everted feet were also observed. Gait analysis indicated short stride (91 cm), slow speed (87 cm/s), poor symmetry, high variability, heel/flat-foot contact, lateral pelvic shift, and increased medial foot pressure.

### Functional assessments

The Berg Balance Scale score declined slightly from 46 (Aug 2024, low fall risk) to 44 (Dec 2024), suggesting emerging dynamic balance issues (Table 1). Although the Gross Motor Function Measure (GMFM-88) remained stable, with a consistent total score of 96.2, residual limitations were observed in standing (Dimension D: 92.3) and advanced locomotion (Dimension E: 88.9) (Table 1). Fine motor function, as measured by the Fine Motor Function Measure (FMFM), exhibited mild bilateral impairment (Grade I) and asymmetric changes (Table 1). Left-hand performance declined slightly from 86.81% to 84.06%, while right-hand performance improved from 83.18% to 86.81%. The Wechsler Intelligence Scale for Children-Fourth Edition (WISC-IV) indicated mild cognitive impairment (full-scale IQ: 74) (Table 2). Marked deficits were noted in perceptual reasoning (score: 60) and processing speed (score: 71).

**Table 1:** Functional assessments

Date	BBS	GMFM-88						FMFM	
		A	B	C	D	E	Total score	Left hand (grade)	Right hand (grade)
August 2024	46	100	100	100	92.3	88.9	96.2	86.81% (I)	83.18% (I)
December 2024	44	100	100	100	92.3	88.9	96.2	84.06% (I)	86.81% (I)

*BBS* Berg Balance Scale, *A* Lying and rolling, *B* sitting, *C* crawling and kneeling, *D* standing, *E* walking, running, and jumping, *GMFM-88* Gross Motor Function Measure-88, *FMFM* Fine Motor Function Measure

**Table 2:** Intelligence quotient results

Scale	Score	Percentile rank	95% CI
Verbal comprehension	83	13	77–91
Perceptual reasoning	60	0.4	56–71
Working memory skills	97	42	90–104
Processing speed	71	3	66–84
Full-scale IQ	74	4	70–80

*CI* confidence interval

### Brain MRI findings

**Fig. 1:**
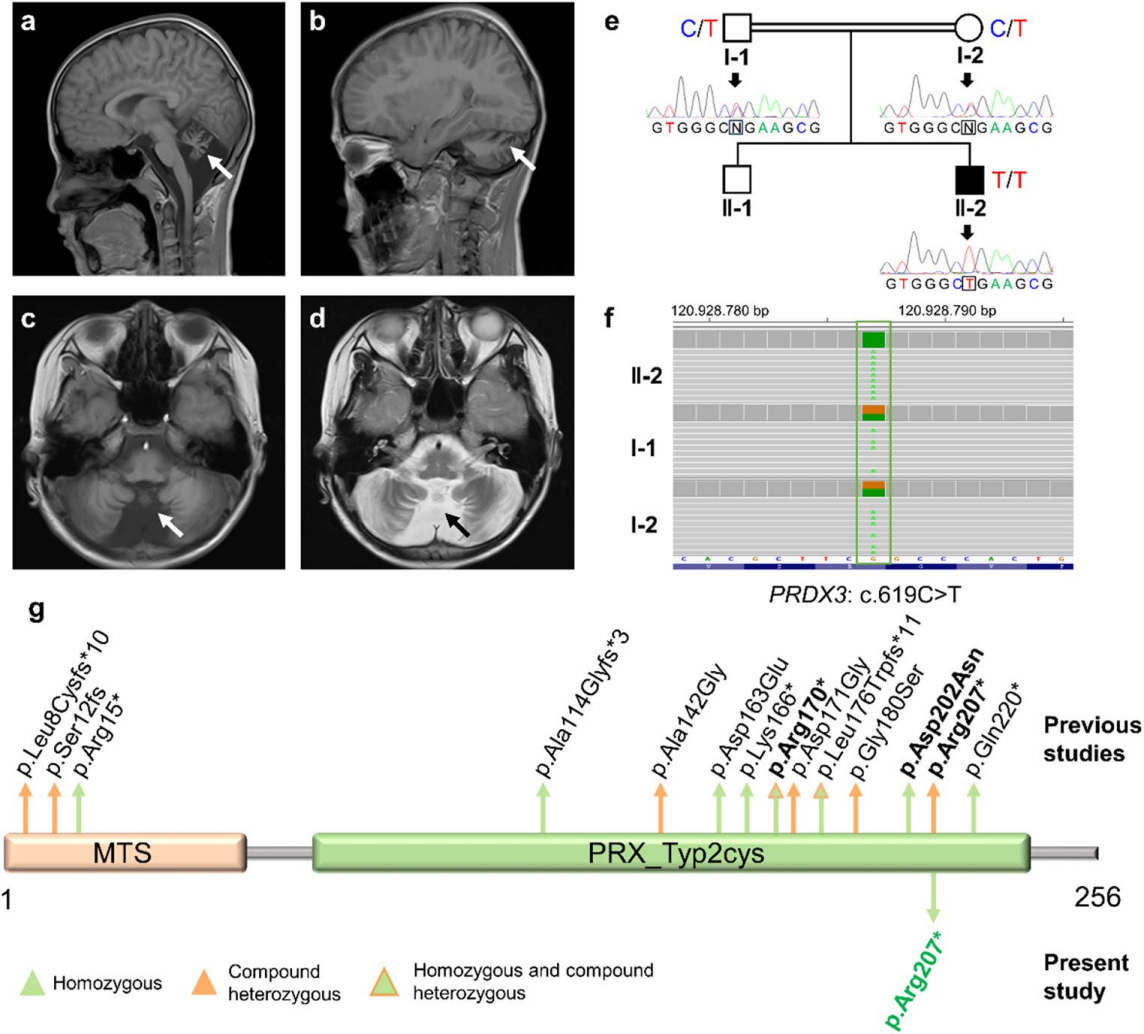
A homozygous pathogenic variant in PRDX3. (**a**-**d**) Brain magnetic resonance imaging (MRI) showed cerebellar atrophy marked with arrows. A and B, sagittal T1 images; C, axial T1-weighted imaging; D, axial T2-weighted imaging. (**e**) Pedigree of the proband (II-2) and Sanger sequencing DNA chromatograms in the family. N denotes the heterozygous C/T allele. (**f**) Variant details of the proband (II-2). (**g**) Previously reported and present studies’ (green) findings on PRDX3 protein level changes. Bold font indicates high-frequency mutations that have been well-documented in at least three patients. MTS, mitochondrial targeting sequence; PRX_Typ2cys, peroxiredoxin, typical 2-Cys type.ssssss

The patient showed extensive cerebellar atrophy on T1- or T2-weighted magnetic resonance imaging (MRI) of the brain (Fig. 1a-d). Specifically, T2 hyperintensities were noticed at both the anterior and posterior cerebellar vermis.

### A homozygous pathogenic variant in PRDX3

Whole-exome sequencing (WES) identified a homozygous nonsense mutation, c.619 C >T (p.Arg207Ter), in exon 6 of the *PRDX3* gene (NM_006793.5), located at chr10:120928787 (Fig. 1e and f). Both parents were heterozygous carriers, as verified by Sanger sequencing, confirming autosomal recessive inheritance (Fig. 1e and f). This rare variant (gnomAD allele frequency: 2/251,446; 1/18,394 East Asian) was predicted to cause premature termination and loss of protein function (Fig. 1g). According to ACMG guidelines, c.619 C >T (p.Arg207Ter) meets PVS1 (null variant), PM2 (rare in population), PP1 (co-segregation), and PP4 (phenotype) criteria, classifying it as a pathogenic variant (Class 5) [4].

### Laboratory measurements

Comprehensive laboratory tests showed decreased levels of selenium (85.44 µg/L), increased levels of thyroid autoantibodies, including anti-thyroid peroxidase (30.54 IU/mL) and anti-thyroglobulin (238.21 IU/mL). These findings were associated with an enlargement and heterogeneous echogenicity of the thyroid gland.

## Discussion

The current study reported a paediatric patient harbouring the homozygous c.619 C >T (p.Arg207Ter) variant, thereby expanding the genotypic and phenotypic spectrum of SCAR32. The patient presented with early-onset progressive gait ataxia and cerebellar atrophy, consistent with SCAR32, but exhibited milder symptoms compared with previously reported cases [2, 2, 5–10]. Notably, he also had mild cognitive impairment (especially in perceptual reasoning and processing speed), elevated thyroid autoantibodies, and thyroid enlargement, suggesting phenotypic variability.

Homozygous and compound heterozygous mutations in the *PRDX3* gene have been previously reported across different ethnic populations (Table 3; Fig. 1g). While the specific c.619 C > T variant has been reported heterozygously in compound genotypes in Korean and Chinese patients (often with more severe features like dysarthria), this was the first documented homozygous occurrence (Table 3).

**Table 3:** Clinical comparison of our case with previously reported cases [2, 3], 5–9– [10]

	Rebelo et al. 2021					Martinez-Rubio et al. 2022	Rafeeq et al. 2022	Rebelo et al. 2022		Efthymiou et al. 2023			Cho et al. 2023		Wang G et al. 2023	Naef et al. 2024		Present study
Case	1	2	3	4	5	1	1	1	2	1	2	3	1	2	1	1	2	1
Ethnicity	Kurdish	Kurdish	German	Indian	French	Moroccan	Pakistani	Syrian	Turkish	Brazilian	Brazilian	Indian	Korean	Korean	Chinese	NM	NM	Chinese
Sex	Male	Male	Female	Male	Male	Male	Female	Female	Male	Male	Male	Male	Male	Female	Male	Male	Male	Male
Age at onset (years)	22	13	23	21	15	<2	<2	2	35	18	35	0.5	25	11	14	10	Early teens	10
Consanguinity	NM	Yes	NM	Yes	No	Yes	Yes	Yes	Yes	No	No	Yes	No	No	No	NM	No	Yes
Nucleotide change	c.604G >A	c.604G >A	c.340dupG	c.508 C >T	c.425 C >G c.37–2 A >G	c.489 C >G	c.496 A >T	c.43 C >T	c.658 C >T	c.604G >A	c.23delT c.538G >A	c.508 C >T	c.508 C >T c.512 A >G	c.508 C >T c.619 C >T	c.619 C >T c.311 + 5G >T	c.525_535delGTTAGAAGGTT	c.525_535delGTTAGAAGGTT c.425 C >G	c.619 C >T
Protein change	p.Asp202Asn	p.Asp202Asn	p.Ala114Glyfs*3	p.Arg170*	p.Ala142Gly p.Ser12fs	p.Asp163Glu	p.Lys166*	p.Arg15*	p.Gln220*	p.Asp202Asn	p.Leu8Cysfs*10 p.Gly180Ser	p.Arg170*	p.Arg170* p.Asp171Gly	p.Arg170* p.Arg207*	p.Arg207* LOF mutation	p.Leu176Trpfs*11	p.Leu176Trpfs*11 p.Ala142Gly	p.Arg207*
Mode of inheritance	HOM	HOM	HOM	HOM	CH	HOM	HOM	HOM	HOM	HOM	CH	HOM	CH	CH	CH	HOM	CH	HOM
Gait ataxia	+	+	+	+	+	+	+	+	+	+	+	+	+	+	+	+	+	+
Limb ataxia	+	+	+	+	+	+	+	+	+	+	+	+	+	+	+	+	+	+
Dysarthria	+	+	+	+	+	+	+	-	+	+	+	+	+	+	+	+	+	-
Oculomotor signs	+	+	+	+	+	+	+	+	+	+	+	+	-	-	+	+	-	-
Myoclonus	+	-	-	-	+	-	-	-	-	-	-	-	-	-	-	-	-	-
Cognitive impairment	-	-	-	-	+	-	+	-	-	-	-	-	-	+	-	+	+	+
Hearing impairment	-	-	-	-	-	-	+	-	-	-	-	-	-	-	-	-	-	-
SARA	14	13.5	21.5	8.5	15	19	10.5	7.5	12	12	12	8	6	8	NA	7	10	NA
MRI (cerebellar atrophy)	+	+	+	+	+	+	+	+	+	+	+	+	+	+	+	+	+	+

*NM* not mentioned, *LOF* loss of function, *HOM* Homozygous, *CH* Compound heterozygous, *FSIQ* full-scale intelligence quotient, *SARA* scale for the assessment and rating of ataxia at the last exam, *NA* not assessed

RNA Fluorescence in situ hybridization (FISH) data from the Allen Brain Atlas (https://mouse.brain-map.org/experiment/show?id=70743842) revealed that the *Prdx3* gene is widely expressed in the cerebellum and hippocampus of mice, indicating its neuronal relevance. PRDX3 protein maintains intracellular redox homeostasis by scavenging reactive oxygen species (ROS). Nonsense mutations in the *PRDX3* gene may result in the depletion of PRDX3 protein, thereby rendering neurons more vulnerable to oxidative stress damage.

Recent studies [11, 12] also implicated PRDX3 in ferroptosis, an iron-dependent form of cell death driven by lipid peroxidation, which leads to PRDX3 hyperoxidation. The hyperoxidized PRDX3 relocates to the plasma membrane and suppresses cystine uptake, thereby promoting ferroptotic cell death. PRDX3 also suppresses lipid ROS accumulation, and its deficiency sensitizes cells to ferroptosis [11, 12]. These mechanisms may contribute to neurodegeneration in *PRDX3*-related SCAR32.

Consequently, all patients exhibited gait and limb ataxia along with cerebellar atrophy (Table 3), albeit with onset ages ranging from birth to 35 years. Additionally, some patients displayed brainstem T2 hyperintensities or olivary degeneration. Notably, consistent with some previous studies, our patient exhibited mild cognitive impairment; however, we did not observe any structural changes in cognition-related brain regions such as the hippocampus and prefrontal cortex. These diverse clinical manifestations underscore the importance of early assessment for cognitive and other related behavioral changes, even in the absence of overt pathological changes.

Additionally, the patient exhibited an increase in anti-thyroid peroxidase and anti-thyroglobulin antibodies, along with thyroid gland enlargement. A previous study demonstrated that dysfunction of PRDX3 impairs mitochondrial antioxidant defence and promotes the accumulation of ROS [2]. This oxidative stress may initiate two key pathological processes: accumulation of ROS in thyroid cells triggers pro-inflammatory pathways (NF-κB, NLRP3 inflammasome) and enhances presentation of thyroid autoantigens. These mechanisms may contribute to the development of thyroid autoimmunity in the context of PRDX3-related pathology. Although thyroid autoimmunity and gland enlargement were observed, a causal association between PRDX3 deficiency and thyroid pathology should be further studied.

This report had several limitations. Firstly, standardized ataxia scales (SARA, FARS-ADL) were not used; paediatric rehabilitation scales (BBS, GMFM-88, FMFM) were applied instead, which provide complementary but non-equivalent indices of motor performance. Future follow-up should include standardized ataxia scales for longitudinal comparison. Secondly, coronal MRI images were unavailable for further assessment although sagittal and axial sequences sufficiently revealed the characteristic cerebellar atrophy.

In conclusion, this study identified a homozygous pathogenic variant in *PRDX3* associated with SCAR32 in a paediatric patient. Although the variant has been previously reported in a heterozygous state, the patient exhibited atypical phenotypes, expanding the knowledge of its clinical spectrum. Furthermore, the findings underscore the need to assess and treat other manifestations, such as cognitive function and thyroid function, at the early stage of the disease onset.

## Data Availability

No datasets were generated or analysed during the current study.
